# CD169-positive macrophages in lymph nodes and the tumor microenvironment: harnessing their biology for clinical diagnosis and anticancer therapeutic strategies

**DOI:** 10.3389/fimmu.2026.1852700

**Published:** 2026-06-24

**Authors:** Ryo Fukuda, Yukio Fujiwara, Toru Maruyama, Yoshihiro Komohara

**Affiliations:** 1 Department of Cell Pathology, Graduate School of Medical Sciences, Faculty of Life Sciences, Kumamoto University, Chuo-ku, Kumamoto, Japan; 2 Department of Biopharmaceutics, Graduate School of Pharmaceutical Sciences, Kumamoto University, Chuo-ku, Kumamoto, Japan

**Keywords:** cancer immunology, cancer immunotherapy, CD169, diagnostic biomarker, lymph node, macrophage, tumor microenvironment

## Abstract

CD169^+^ macrophages are strategically positioned in secondary lymphoid organs and diverse peripheral tissues, where they orchestrate tissue-specific immune homeostasis by regulating local cellular and humoral responses. In the context of cancer, CD169^+^ macrophages within tumor-draining lymph nodes (tdLNs) and the tumor microenvironment (TME) have emerged as critical determinants of clinical outcome across multiple malignancies, acting as key modulators of both tumor progression and tumor control. In this review, we delineate the distinct yet complementary roles of CD169^+^ macrophages in tdLNs and the TME, with particular emphasis on their molecular and cellular interactions with cancer cells and immune effector populations, and on how these interactions shape systemic anticancer immunity. We further discuss the translational significance of CD169^+^ macrophages as prognostic and predictive biomarkers, and propose immunotherapeutic strategies that selectively target or exploit these cells to enhance antitumor responses. By integrating current experimental and clinical evidence, this review aims to refine our understanding of CD169^+^ macrophage biology and to highlight their potential as actionable targets in cancer diagnosis and treatment.

## 1 Introduction

CD169^+^ macrophages represent a functionally specialized macrophage subset that cannot be adequately captured by the conventional M1/M2 dichotomy (1). While classically described in lymphoid organs—including lymph nodes (LNs) and spleen (2, 3)—CD169^+^ macrophages are now recognized as constitutive residents in a broad spectrum of non-lymphoid tissues, such as the lung (4–6), liver (7–9), brain (10, 11), skin (12, 13), Osseous tissue (14–16), adipose tissue (17), kidneys (18, 19), and colon (20, 21), where they contribute to tissue-specific immune surveillance and homeostatic regulation. Mechanistically, recent studies have shown that surface CD169 can facilitate binding and internalization of SARS-CoV-2 (22, 23). In parallel, pulmonary CD169^+^ macrophages have been implicated in host protection by limiting viral spread and restraining excessive inflammation (24). Collectively, these advances position CD169^+^ macrophages as an emerging therapeutic node in immune-related diseases.

In oncology, expanding interrogation of the immune landscape has highlighted pivotal roles for CD169^+^ macrophages not only within the tumor microenvironment (TME) but also in tumor-draining LNs (tdLNs), a central immunological hub for priming and shaping antitumor immunity. However, the functional consequences of CD169^+^ macrophages across these compartments—and their value as prognostic biomarkers—appear highly context-dependent and, in some settings, seemingly contradictory. Such divergence likely reflects heterogeneity in tissue niche, tumor type, immunogenicity, and treatment context, underscoring the need for a compartment-specific and mechanism-informed framework to interpret their biology and therapeutic relevance.

In this review, we delineate the intricate interplay between CD169^+^ macrophages and their tumor-promoting or suppressive functions across the TME and tdLN, while also highlighting their clinical significance as prognostic indicators. By further unveiling therapeutic strategies centered on these cells, this work aims to catalyze advancements in tumor immunology, enhance diagnostic precision, and accelerate the trajectory of novel drug discovery.

## 2 Origin and maintenance of CD169^+^ macrophages in LNs

The origin of tissue-resident macrophages is characterized by remarkable complexity, involving contributions from both embryonic precursors—originating in the yolk sac and fetal liver—and bone marrow-derived monocytes that emerge postnatally from hematopoietic stem cells (HSCs). For example, microglia and Kupffer cells are established during embryogenesis and persist without substantial replacement by HSC-derived cells throughout life (25, 26). In contrast, embryonically seeded macrophages in the colon are gradually supplanted by HSC-derived monocytes after birth (27). Other tissues, such as the pancreas, lung, and heart, display a mosaic macrophage composition of both embryonic and postnatal origins (28–30).

In recent years, the developmental origin and maintenance mechanisms of LN-resident macrophages—particularly CD169^+^ subset—have garnered increasing attention. Mondor et al. employed sophisticated genetic fate-mapping and intravital imaging techniques to demonstrate that subcapsular sinus (SCS) CD169^+^ macrophages are initially derived from yolk sac progenitors during early embryogenesis. However, these embryonic macrophages are largely replaced postnatally by HSC-derived monocytes, which subsequently undergo local proliferation within the LN microenvironment. Their maintenance is critically dependent on colony-stimulating factor 1 (CSF-1) secreted by neighboring lymphatic endothelial cells. This niche dependency extends to medullary sinus macrophages, which also express CD169 (31). Furthermore, Camara et al. reported that the differentiation of SCS CD169^+^ macrophages indispensably requires coordinated and direct stimulation by both receptor activator of NF-κB ligand (RANKL) produced by marginal reticular cells (MRCs) and lymphotoxin-αβ (LTαβ) produced by B cells (32). Additionally, D’Addio et al. demonstrated that densely clustered sialylated glycans on the floor of the SCS interact with CD169 on the macrophages, contributing to their survival and functional maintenance (33). Thus, a diverse range of cells within the LN contributes to the maintenance and niche formation for CD169^+^ macrophages.

Collectively, these findings underscore that the LN microenvironment orchestrates the origin, maintenance, and specialization of CD169^+^ macrophages through a dynamic interplay of cellular interactions and molecular signaling. This tightly regulated niche is essential for sustaining the unique sentinel and immunoregulatory roles of these macrophages at the lymph-tissue interface.

## 3 The clinical and biological significance of CD169^+^ macrophages in tdLNs of solid tumors

### 3.1 Clinical significance of CD169^+^ macrophages as prognostic determinants in solid tumors

TdLNs, which are directly connected to the primary tumor via lymphatic vessels, play pivotal roles in both the metastatic dissemination of cancer and the modulation of antitumor immunity. Consequently, the immunological landscape of tdLNs is intimately linked to tumor progression and clinical staging. Among the immune constituents of tdLNs, CD169^+^ macrophages have emerged as a particularly noteworthy population due to their pro-inflammatory and immunostimulatory functions, with potential to enhance antitumor immunity (34). Intriguingly, multiple clinical studies have demonstrated that high densities of CD169^+^ macrophages within tdLNs are significantly associated with improved overall survival (OS) or progression-free survival in patients with a variety of solid tumors, including colorectal cancer (35), melanoma (36), endometrial cancer (37), bladder cancer (38), esophageal cancer (39), breast cancer (40, 41), gastric cancer (42), oral squamous cell carcinoma (43), and prostate cancer (44). In the majority of these contexts, elevated CD169^+^ macrophage levels positively correlate with increased infiltration of intratumoral CD8^+^ T lymphocytes, suggesting their role in shaping effective antitumor immune responses.

### 3.2 Determinants of CD169^+^ macrophage dynamics in tdLNs

The mechanisms underlying the variable abundance of CD169^+^ macrophages in tdLNs—even among patients with the same cancer type—remain incompletely understood (45). Maeshima et al. reported that CD169^+^ macrophages are consistently diminished in metastatic LNs across the three major subtypes of breast cancer (luminal, human epidermal growth factor receptor type2 (HER2)-positive, and triple-negative) (46). Complementing this, Briem et al. used spatial proteomics to reveal a marked reduction of subcapsular sinus-resident CD169^+^ macrophages upon nodal metastasis, alongside a notable downregulation of the anti-apoptotic protein B cell lymphoma-extra-large (Bcl-xL) (47). Furthermore, our own studies in prostate and colorectal cancer cohorts have suggested that host factors such as aging may contribute to the decline of CD169^+^ macrophage populations in tdLNs (48, 49). These observations collectively underscore the need for deeper mechanistic insights into how metastatic cancer cells interact with CD169^+^ macrophages, as well as how host-related variables—such as age, immune status, or metabolic factors—may influence their maintenance. Such knowledge could prove instrumental in devising strategies to preserve or enhance CD169^+^ macrophage function in the context of cancer immunotherapy.

Given that tumor cell characteristics and the surrounding TME vary considerably among cancer types, the frequency and functionality of CD169^+^ macrophages are also likely to differ accordingly. Supporting this, our murine studies using subcutaneous tumor models revealed a significant reduction in CD169 expression within tdLNs of mice bearing MB49 or LLC tumors—models typically categorized as immunologically “cold” tumors (50–52) —compared to those bearing MC38 or E0771 tumors, which represent “hot” tumor models (53–55). Whether such differences stem from intrinsic tumor cell factors or extrinsic environmental cues within the TME remains to be elucidated through broader comparative analyses across tumor types.

Importantly, tdLNs are not only sites of metastatic cell invasion but are also exposed to a milieu of tumor-derived soluble factors, including cytokines, exosomes, and antigens (56). Therefore, the identification of molecular or cellular mediators that suppress CD169 expression within tdLNs may reveal novel immunosuppressive pathways. Targeting these pathways could restore or augment the function of CD169^+^ macrophages and thereby potentiate antitumor immunity, offering a promising avenue for therapeutic intervention.

## 4 The multifaceted functions of LN CD169^+^ macrophages in murine antitumor immunity

### 4.1 CD169^+^ macrophages as pivotal antigen presenting cells for CD8^+^ T cell priming

LN-resident CD169^+^ macrophages have emerged as pivotal regulators of antitumor immune responses, exerting control over both innate and adaptive immunity. Asano et al. were the first to delineate the *in vivo* mechanism underlying their immunostimulatory function in 2011—preceding numerous clinical studies that later underscored their prognostic relevance in various malignancies. These macrophages reside primarily in the subcapsular and medullary sinuses of the tdLNs and recognize apoptotic tumor cells via surface phosphatidylserine, thereby enabling efficient antigen uptake. A subset of these CD169^+^ macrophages co-expressing CD11c, located at the T cell/B cell interface, subsequently cross-presents tumor antigens to naïve CD8^+^ T cells, initiating cytotoxic T lymphocyte responses (57). Notably, CD169^+^ macrophages residing in the subcapsular sinus of LNs have been shown to capture and present tumor-derived antigens to CD8^+^ T cells via cross-presentation, thereby acting as a crucial bridge between innate and adaptive immunity. This function may underlie the observed correlation between high CD169^+^ macrophage density and increased intratumoral CD8^+^ T cell infiltration. Beyond their classical antigen presenting functions, CD169 itself may play a more direct role in modulating T cell activity (58, 59). CD43, a sialylated glycoprotein expressed on T cells, has been identified as a putative counter-receptor for CD169 (60). Although the precise contribution of CD169–CD43 interactions to T cell priming in cancer remains to be fully elucidated, it is notable that this molecular engagement enhances intercellular adhesion (61) and that CD43 signaling can augment T cell activation (62, 63). These findings collectively support the hypothesis that CD169 may deliver co-stimulatory signals that facilitate robust activation of tumor antigen-specific CD8^+^ T cells.

### 4.2 Potential crosstalk between CD169^+^ macrophages and NK cells

The critical role of subcapsular CD169^+^ macrophages in recruiting and activating NK cells during viral infections has been well established. Specifically, administration of clodronate liposomes reduced both the accumulation and activation of NK cells in the LNs following viral infection. This finding was further corroborated by the result that co-culture with virus-exposed CD169^+^ macrophages led to an increase in activated NK cells (64, 65). However, the precise mechanisms by which these macrophages modulate NK cell activity in the context of cancer remain poorly defined and warrant further investigation.

### 4.3 CD169^+^ macrophages as regulators of B cell responses

Recently, B cells, too, have gained recognition as important players in antitumor immunity, particularly in light of recent findings that underscore their heterogeneity and context-dependent roles (66). Subcapsular CD169^+^ macrophages, positioned adjacent to B cell follicles, are uniquely equipped to capture not only viral particles but also subcellular tumor-derived materials such as exosomes and microvesicles. Crucially, these macrophages subsequently shuttle these intact antigens to follicular dendritic cells, playing a pivotal role in facilitating B cell scanning (67). Tacconi et al. demonstrated in a murine model of breast cancer that these macrophages upregulate genes associated with B cell antigen presentation, thereby promoting B cell activation and potentially mediating anti-metastatic effects in the lung (68). Complementary findings by Pucci et al. showed that CD169^+^ macrophages can sequester tumor-derived exosomes, thereby limiting their immunosuppressive effects on B cells, particularly those subsets known to facilitate tumor progression (69).

**Figure 1:**
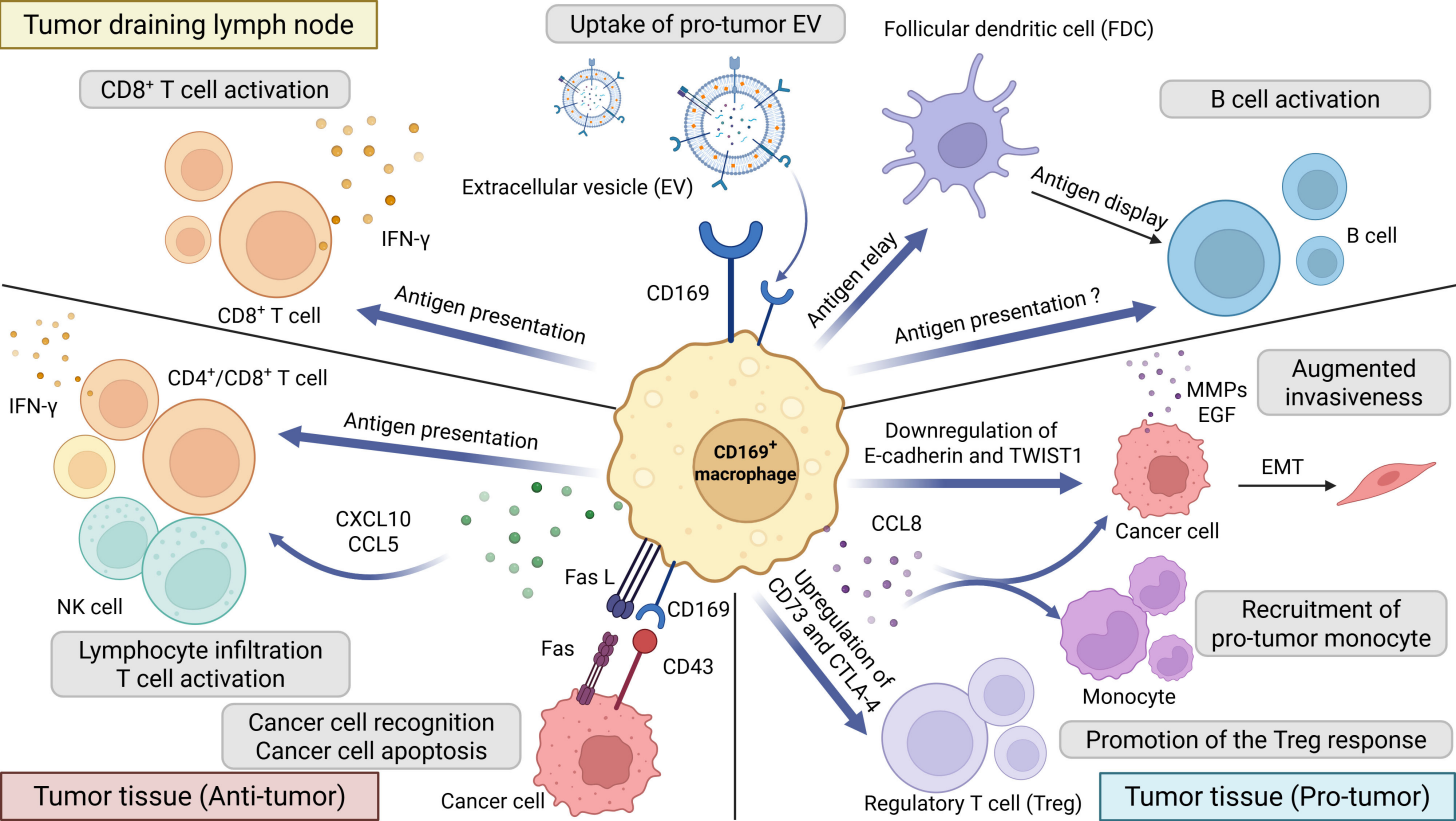
CD169^+^ macrophages as key modulators of tumor immunity in tumor-draining lymph nodes and the tumor microenvironments. In tumor-draining lymph nodes, CD169^+^ macrophages initiate CD8^+^ T cell activation through the uptake of tumor antigens. They also facilitate B cell activation, either via direct antigen presentation or indirectly through follicular dendritic cell (FDC)-mediated pathways. The CD169 molecule itself further contributes to tumor suppression by capturing and clearing tumor-derived extracellular vesicles (EVs). In the tumor microenvironments, CD169^+^ macrophages exhibit a dual functional nature. Their tumor-suppressive functions include orchestrating T cell activation and promoting lymphocyte recruitment through chemokine secretion. Additionally, CD169 molecules directly engage sialic acid ligands such as CD43 on cancer cells, inducing apoptosis via the Fas/FasL signaling axis. Conversely, CD169^+^ macrophages can exert tumor-promoting effects by driving epithelial-mesenchymal transition (EMT) and enhancing cancer invasiveness through the modulation of E-cadherin, TWIST1, matrix metalloproteinases (MMPs), and epidermal growth factor (EGF). Macrophage-derived CCL8 further facilitates both tumor cell invasion and the recruitment of pro-tumorigenic monocytes. Moreover, these macrophages contribute to the establishment of an immunosuppressive microenvironment by upregulating CD73 and CTLA-4 in regulatory T cells (Tregs). Figure was created with BioRender.com.

Taken together, these data highlight LN CD169^+^ macrophages as central orchestrators of antitumor immunity, acting at the nexus of T cell priming, NK cell recruitment, and B cell modulation (**Figure 1**). Their strategic localization, molecular specialization, and functional versatility position them as attractive targets for immunotherapeutic intervention aimed at enhancing LN-mediated immune surveillance and tumor control.

## 5 Context-specific origin and induction of CD169^+^ macrophages in the murine TME

Tumor-associated macrophages (TAMs) comprise a highly plastic and phenotypically heterogeneous myeloid compartment within the TME. From a developmental perspective, they can be broadly subdivided into macrophages derived from circulating monocytes and those originating from embryonic progenitors (yolk sac/fetal liver), with local microenvironmental cues dictating their polarization state and functional reprogramming (70). In the GL261 murine glioma model, the intratumoral CD169^+^ macrophage subset has been shown to arise predominantly from monocyte-derived infiltrates, and its acquisition of CD169 is critically dependent on interferon-γ (IFN-γ) signaling provided by NK cells (71). A conceptually similar, monocyte-derived trajectory is also reported in hepatocellular carcinoma (HCC), although the upstream inputs differ: in this setting, type I interferons secreted by multiple leukocyte populations—including CD4^+^ and CD8^+^ T cells—potentiate CD169 expression on monocytes (72). In contrast, in non-small cell lung cancer (NSCLC), elevated CD169 expression is preferentially observed in tissue-resident alveolar macrophages rather than in monocyte-derived macrophages, and these resident CD169^+^ macrophages have been implicated in promoting early tumor progression—highlighting lineage-dependent functional divergence in a tissue-specific TME (73).

Beyond immune cell–mediated induction, hypoxia has emerged as an additional and potentially indirect regulatory axis governing CD169 expression. *In vitro* studies have demonstrated that hypoxic macrophages activate hypoxia-inducible factor-1α (HIF-1α), thereby promoting IFN-γ production, enhancing phagocytic activity, and upregulating activation-associated molecules, including CD40, CD86, and MHC class I (74). In line with these observations, monocytes exposed to hypoxic conditions exhibit increased CD169 expression (75). Nevertheless, it remains unclear whether hypoxia-driven augmentation of CD169 is mediated predominantly through macrophage-intrinsic IFN-γ signaling or through direct transcriptional regulation by hypoxia-responsive pathways such as HIF-1α. Addressing this issue will require mechanistic studies incorporating hypoxic stimulation under IFN signaling blockade, together with direct interrogation of HIF-1α occupancy and transcriptional activity at hypoxia response element–containing regions of Siglec1 (CD169) using chromatin immunoprecipitation and reporter assays. This regulatory complexity is compounded by additional TME-derived constraints and countervailing signals: antigen-specific CD8^+^ T cells can exhibit diminished IFN-γ production under hypoxia (76); transforming growth factor-β (TGF-β) suppresses macrophage CD169 expression (59); and inflammatory cytokines such as IL-6 and IL-1β may dampen mTORC1 activity, potentially repressing C/EBPα, a transcription factor implicated in CD169 expression (77).

Collectively, these observations support a model in which CD169 expression is shaped by the integration of spatially and temporally heterogeneous signals—oxygen tension gradients, the proximity of macrophages to IFN-producing lymphocytes, and cytokine-mediated crosstalk—underscoring the need for region-resolved, spatially informed analyses to delineate the dominant mechanisms controlling CD169 induction within the TME.

## 6 Clinical significance of intratumoral CD169^+^ macrophages in human solid tumors

CD169^+^ macrophages in tdLNs are well-established prognostic correlates across multiple malignancies. Accumulating evidence now indicates that CD169^+^ macrophages residing within the TME may also convey clinically meaningful information, although the direction and magnitude of their prognostic impact appear to be tumor type– and context-dependent.

### 6.1 CD169^+^ macrophages as a favorable prognostic marker

In glioma, patients with a high intratumoral CD169/CD68 ratio exhibit superior OS and show increased expression of immune activation signatures, including CD3E, NCR1, and IFN-γ, compared with those with a low ratio (71). Concordantly, in HCC and gastric cancer, a high density of intratumoral CD169^+^ macrophages is associated with improved OS and positively correlates with the abundance of intratumoral CD8^+^ T cells (72, 78). In HCC, these associations have been further supported by a meta-analysis demonstrating a significant relationship between CD169^+^ macrophage infiltration in the tumor interior and OS, reinforcing the concept that this population may serve as a prognostic biomarker (79). Similar trends have been reported in colorectal cancer, where a higher proportion of CD169^+^ macrophages correlates with improved OS and an inverse association with TNM stage (80). In pancreatic ductal adenocarcinoma (PDAC), refined phenotyping of TAMs has also provided supportive evidence: CD169 is enriched in a “favorable” bM2-like TAM subset relative to an “unfavorable” mM2-like subset, suggesting that CD169 expression may demarcate prognostically beneficial macrophage programs even within M2-like compartments (81).

### 6.2 CD169^+^ macrophages as an unfavorable prognostic marker

Notably, however, the prognostic relevance of intratumoral CD169^+^ macrophages is not uniformly favorable. In bladder cancer, intratumoral CD169^+^ macrophage density does not significantly stratify cancer-specific survival, and the high-density group exhibits increased lymphovascular invasion, raising the possibility of a context-dependent deleterious association (82). In breast cancer, the presence versus absence of CD169^+^ macrophages is not significantly associated with distant recurrence-free interval (41), and transcript-level analyses further complicate interpretation: high SIGLEC1 expression in the METABRIC cohort has been linked to reduced disease-specific survival (83).

These discordant observations underscore that, unlike the relatively consistent associations seen in LNs, the prognostic meaning of CD169 within tumors is shaped by local ecology—cellular origin (resident vs monocyte-derived), coexisting cytokine milieus, spatial distribution, and co-expression of immunoregulatory molecules. Given the profound heterogeneity of the TME and the recognized limitations of the M1/M2 framework, CD169 should be viewed less as a universal “good macrophage” marker and more as a contextual indicator of specific macrophage states that can diverge across tumor types. Accordingly, translation of intratumoral CD169^+^ macrophages into robust diagnostic or prognostic tools will require higher-resolution subclassification (e.g., multiplex IHC/spatial transcriptomics and single-cell profiling), harmonized scoring strategies, and validation in large, well-annotated cohorts and meta-analyses. Importantly, because multiple studies have reported a positive association between CD169^+^ macrophage density and CD8^+^ T cell infiltration, intratumoral CD169^+^ macrophages are attractive not only as prognostic correlates but also as candidate biomarkers for predicting responsiveness to immune checkpoint blockade, particularly in settings where they reflect an IFN-licensed, T cell–permissive immune contexture. On the other hand, multiple studies have also reported that the frequency of CD169^+^ macrophages within the TME is generally low. Accordingly, the use of CD169 as a diagnostic marker in tumor specimens necessitates stringent validation to ensure analytical accuracy.

## 7 Functional roles of intratumoral CD169^+^ macrophages in tumor immunity

Reflecting the heterogeneous prognostic associations of intratumoral CD169^+^ macrophages among cancer types, accumulating evidence indicates that these cells can mediate both tumor-suppressive and tumor-promoting activities depending on context (**Figure 1**).

### 7.1 Antitumor functional roles of CD169^+^ macrophages in murine models

With respect to antitumor immunity, studies using the GL261 murine glioma model showed that CD169^+^ macrophages display markedly elevated expression of interferon-stimulated genes (ISGs) relative to CD169^-^ counterparts, culminating in robust intratumoral production of the T cell–recruiting chemokines CXCL10 and CCL5. Conversely, depletion of CD169^+^ macrophages attenuated chemokine production and impaired both the recruitment and effector functionality of tumor-infiltrating T cells. In addition to shaping leukocyte trafficking, CD169 has been reported to directly recognize sialylated glycoproteins and/or glycolipids on GL261 cells, thereby facilitating macrophage-mediated phagocytosis (71). Collectively, these data support a model in which CD169^+^ macrophages reinforce antitumor immunity by coordinating immune cell recruitment while promoting antigen uptake and presentation within the TME. A complementary tumoricidal function of the CD169 axis has been described in the MC38 murine colorectal cancer model, where CD169 was identified to engage CD43 on cancer cells and induce apoptosis through the Fas receptor/Fas ligand pathway (80). Consistent with this mechanism, cancer cells engineered to express high-affinity Siglec-1 ligands undergo cell death upon co-culture with CD169^+^ macrophages (84). Together, these findings underscore the capacity of CD169-dependent interactions to directly trigger tumor cell elimination and to mechanistically bridge innate effector functions with downstream adaptive immune responses. Notably, several CD169 ligands—including CD43, mucin-1 (MUC1) (85), and gangliosides (86)—are broadly overexpressed across malignancies and have been implicated in tumor growth, metastasis, and remodeling of the TME (87–92). This raises the possibility that the CD169–sialic acid ligand axis is not merely a recognition module for macrophages but may also influence intrinsic cancer cell signaling programs, thereby contributing to growth restraint and/or programmed cell death in ligand-dependent contexts.

### 7.2 Pro-tumor functional roles of CD169^+^ macrophages

In contrast, pro-tumorigenic functions of intratumoral CD169^+^ macrophages have also been reported.

#### 7.2.1 Murine models

NSCLC models suggest that CD169^+^ tissue-resident macrophages can modulate epithelial–mesenchymal transition (EMT)-related factors in cancer cells—such as TWIST1 and E-cadherin—and promote a regulatory T cell (Treg) response characterized by the induction of CD73 and CTLA-4 expression, which protects cancer cells from attack by CD8^+^ T cells (73).

#### 7.2.2 Human cancers

Cassetta et al. demonstrated that CD169^+^ tumor-associated macrophages (TAMs) in breast cancer promote malignancy through CCL8 production, which enhances cancer cell motility, induces invasion- and progression-associated gene programs, and facilitates monocyte recruitment into the TME (83). Moreover, Gunnarsdottir et al. demonstrated that IFN-α–induced CD169^+^ macrophages display a distinct immunoregulatory phenotype characterized by elevated expression of mediators such as PGE2, HLA-G, and IL-10, in contrast to LPS/IFN-γ–polarized M1-like macrophages and IL-4–induced M2-like macrophages, both of which exhibit minimal to absent CD169 expression. Functional co-culture assays further showed that these CD169^+^ macrophages suppress T cell and NK cell proliferation and activation and diminish antitumor cytotoxicity against MDA-MB-231 breast cancer cells to an extent comparable to M2-like macrophages (93). Intriguingly, the same study interrogated the relationship between intratumoral CD169^+^ macrophages and tertiary lymphoid structures (TLS) in breast cancer and identified a significant association between CD169^+^ macrophage infiltration and TLS presence. Notably, a subset of tumors characterized by high CD169^+^ macrophage abundance also showed enrichment of gene signatures linked to TLS, regulatory B cells (Bregs), and Tregs (94). Given that TLS can function as intratumoral sites for antigen presentation and lymphocyte priming—and have been associated with improved prognosis and enhanced responsiveness to immune checkpoint blockade (95) —dissecting how CD169^+^ macrophages influence TLS initiation, maturation, and cellular composition is of particular importance. Therefore, defining the context-specific impact of CD169^+^ macrophages on TLS biology and the surrounding immunoregulatory landscape across distinct tumor entities will be critical for refining prognostic stratification and optimizing patient selection for immunotherapy.

## 8 CD169^+^ macrophage-targeted therapeutic strategies for cancer

**Figure 2:**
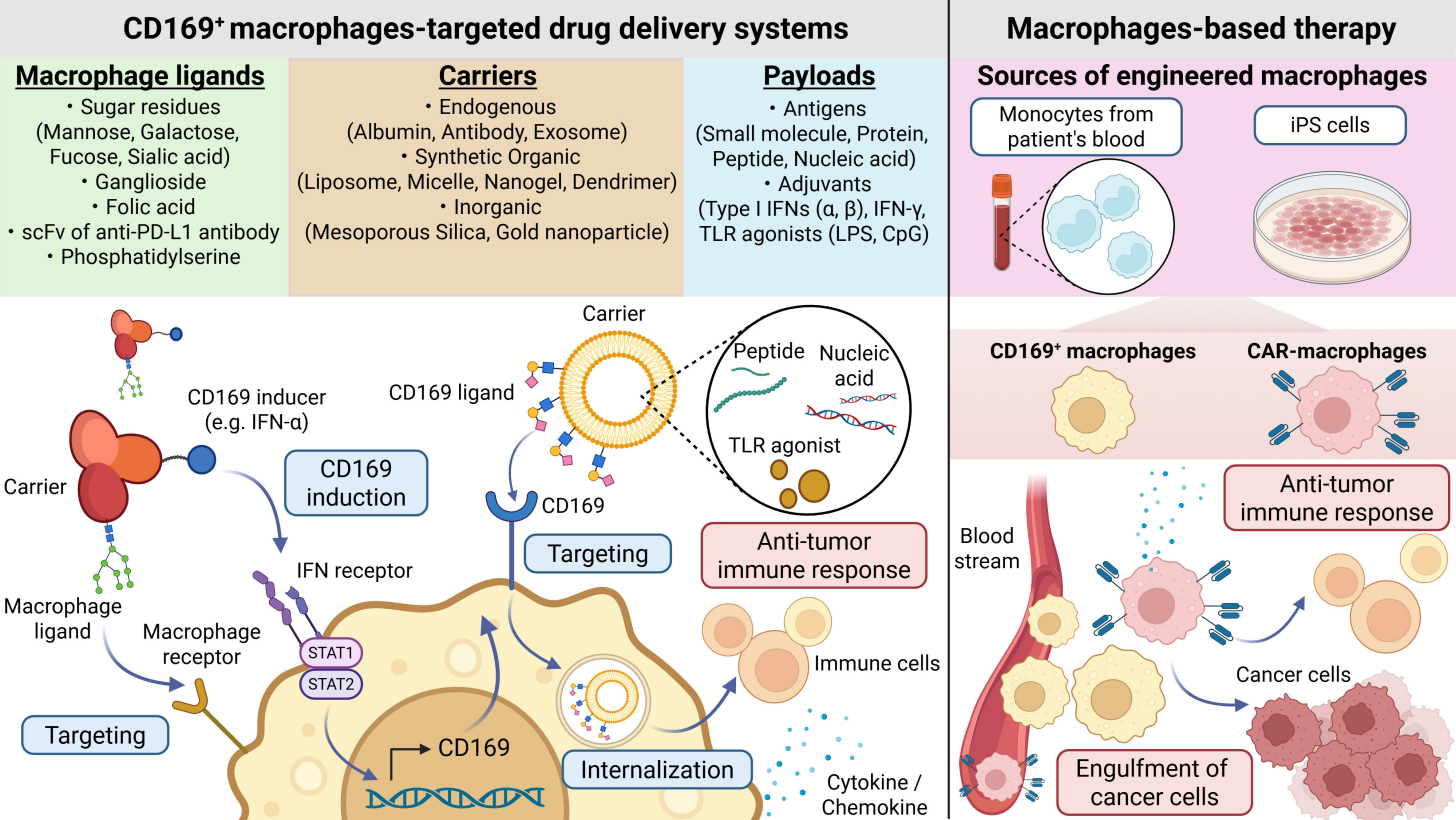
Overview of therapeutic strategies targeting CD169^+^ macrophages in cancer. Targeted delivery of CD169 inducer via macrophage-specific carriers enhances antitumor immunity by upregulating CD169 expression in tumor-draining lymph nodes and the tumor microenvironments. Furthermore, CD169 ligand-coated carriers enable receptor-mediated endocytosis of antigens and adjuvants, resulting in potent antitumor responses through augmented antigen presentation. Next-generation macrophage therapies, utilizing either monocyte-derived or iPSC-derived platforms (e.g., CD169^+^ or CAR-macrophages), effectively amplify antitumor immunity. In the tumor microenvironment, these engineered macrophages promote immune activation via inflammatory cytokine secretion, direct phagocytosis of malignant cells, and the initiation of T cell responses through efficient antigen presentation. Figure was created with BioRender.com.

Given their central role as orchestrators of antitumor immunity, cancer immunotherapies directed at CD169^+^ macrophages—leveraging a variety of biomaterials and engineered macrophage platforms—are currently undergoing intensive development (**Figure 2**).

### 8.1 Induction of CD169^+^ macrophages in LNs

Recently, a novel fusion construct designed to efficiently induce CD169^+^ macrophages within LNs has been reported. This formulation exploits albumin—an abundant component of lymphatic and interstitial fluids —as a carrier, which is decorated with mannosyl chains for macrophage targeting and fused to IFN-α. Following subcutaneous administration, the construct achieves high delivery to tdLNs, leveraging both albumin’s favorable lymphatic transport properties and the mannose-dependent targeting of LN-resident macrophage subsets. This targeted LN delivery robustly induced CD169^+^ macrophages in the tdLNs, enhanced CD8^+^ T cell priming, and culminated in marked suppression of tumor growth (96). Albumin is qualified as a LN-targeted drug delivery vehicle (97) and sentinel LN imaging agent (98, 99) due to its high biocompatibility and fascinating molecular properties for lymphatic transport—66.5 kDa (molecular mass), 10 nm (diameter), and negatively charged surface. Importantly, LN-resident CD169^+^ macrophages typically exhibit high expression of CD206 and other C-type lectins such as CD209 (3, 100). Given that CD206^+^ macrophages in tdLNs often increase in correlation with primary tumor malignancy (101), mannosylated albumin represents a highly attractive carrier for targeting these populations. Consistent with this rationale, mannosylated albumin–based imaging probes exhibit efficient accumulation in tdLNs (102–108).

Beyond direct induction through type I interferons, indirect approaches—such as nanoparticle-mediated delivery of STING or TLR agonists—may also promote CD169 expression. A key caveat, however, is that type I IFNs can, depending on context and dose, drive macrophages toward immunoregulatory or M2-like phenotype (109). For instance, high-dose IFN-α formulation has been reported to act on Kupffer cells to upregulate IL-10 and PD-L1, thereby suppressing Concanavalin A-induced liver injury (110). Furthermore, CD169 expression in tdLNs varies widely among patients, even within the same cancer type, implying inter-individual differences in IFN responsiveness and LN microenvironmental cues. Accordingly, CD169-targeted strategies should be implemented alongside careful immune monitoring, including macrophage polarization states and T cell activation/exhaustion phenotypes. In parallel, the development of liquid-biopsy modalities capable of predicting LN CD169 status would enable patient stratification, real-time pharmacodynamic assessment, and rational personalization of therapy.

### 8.2 Induction of CD169^+^ macrophages in the TME

Because TAMs are abundant in the TME and actively drive malignancy, reprogramming TAMs toward an antitumor, CD169^+^ phenotype state represents a compelling therapeutic approach. Supporting this concept, intravenous administration of an IFN-α–IgG fusion protein in a hepatocellular carcinoma mouse model expanded CD169^+^ macrophages within the TME and significantly inhibited tumor growth (72). Leveraging the high PD-L1 expression frequently observed in TMEs, Liang et al. designed a bifunctional heterodimer that couples a single-chain variable fragment (scFv) of anti-PD-L1 antibody to IFN-α (IFN-α-anti-PD-L1). This agent enhanced intratumoral accumulation of IFN-α and promoted productive engagement of antigen presenting cells, resulting in potent tumor control through amplified antitumor immunity (111). Although CD169 induction was not directly assessed, the mechanistic axis—IFN-α-driven activation of antigen presenting compartments—is highly compatible with the emergence of CD169^+^ macrophage programs. Notably, IFN-α can upregulate PD-L1, potentially creating a reinforcing loop that further increases the retention and activity of IFN-α–anti–PD-L1 within the TME.

Beyond phenotypic reprogramming, CD169^+^ macrophages are linked to chemokine production that facilitates monocyte recruitment into tumors (71, 83). Therefore, strategies that induce CD169^+^ macrophages in the TME may simultaneously enhance both the functional activation of macrophages and the overall abundance of this antitumor subset.

### 8.3 CD169-targeted macrophage delivery systems

Following ligand binding, CD169 can trigger clathrin-mediated endocytosis (112), providing a practical molecular entry point for macrophage-selective drug delivery. CD169 recognizes sialic acid–containing structures, including sialylated glycosphingolipids such as gangliosides (113, 114). Nanoparticles functionalized with these ligands can be internalized in a CD169-dependent manner, enabling delivery of diverse payloads—including small molecules, adjuvants, peptides, proteins, and nucleic acids—and thereby promoting antitumor immune activation (115–120). Recent advances include CD169-specific nanobody–modified liposomes, which can achieve superior internalization efficiency compared with natural ligands such as GM3 (121). Because CD169 induction can increase target density and improve nanoparticle capture, combining CD169-inducing regimens with CD169-targeted vaccines or nanomedicines is a rational strategy to further amplify therapeutic efficacy.

A particularly notable application is the exploitation of the CD169 endocytic pathway for invariant natural killer T (iNKT) cell activation. CD169-targeted liposomes encapsulating α-galactosylceramide (α-GalCer)—a potent glycolipid antigen—robustly primed iNKT cells in the liver and spleen, an effect completely abrogated in CD169-deficient mice (122). These findings indicate that CD169-mediated uptake is not merely a high-capacity internalization route, but a specialized pathway that supports efficient antigen loading onto CD1d for iNKT recognition. Given that iNKT cells can exert direct antitumor activity and coordinate broader innate and adaptive responses (123), CD169-targeted nanoparticles co-delivering tumor antigens, adjuvants, and α-GalCer represent a promising platform for integrated immune potentiation.

### 8.4 Cell-based therapies harnessing CD169^+^ macrophages

Cellular immunotherapies—including CAR-T cells, tumor-infiltrating lymphocytes, and dendritic cell–based approaches—continue to progress through preclinical and clinical development and have demonstrated the capacity to meaningfully improve outcomes in selected settings (124). Macrophages have recently emerged as a compelling cellular therapy modality, and accumulating evidence supports the therapeutic utility of the CD169^+^ subset. For example, Song et al. demonstrated that adoptive transfer of CD169^+^ macrophages enhanced the abscopal effect induced by radiofrequency ablation in hepatic tumors (125), highlighting the ability of this subset to coordinate systemic antitumor immunity. Concurrently, genetic engineering has expanded the scope of macrophage-based therapies, with CAR-macrophages now representing a rapidly advancing frontier; notably, clinical trials in patients with solid tumors are already underway (126). Next-generation CAR-macrophages are being designed not only to enhance phagocytosis but also to enforce or sustain a pro-inflammatory, M1-like state upon antigen engagement, thereby strengthening antitumor efficacy (127). Macrophages offer intrinsic advantages for solid tumors, including tumor homing, tissue infiltration, and the capacity to remodel immunosuppressive TMEs. Nevertheless, key translational challenges remain—such as manufacturing scalability and consistency, durability of antitumor function, and long-term safety—which will require rigorous evaluation in human cohorts and/or humanized model systems.

## 9 Conclusions and perspectives

This review highlights CD169^+^ macrophages as key organizers of tumor immunity across the TME and LNs, with increasing evidence supporting their clinical value as prognostic biomarkers. We also summarize emerging drug delivery concepts designed to preferentially target this subset. Despite these advances, fundamental questions remain regarding the mechanisms that sustain CD169^+^ macrophage identity and function, and how CD169-based readouts can be implemented as reliable, scalable diagnostics.

First, CD169^+^ macrophage abundance in regional LNs varies markedly even among patients with the same malignancy, yet the determinants shaping this population along the TME–LN axis are poorly defined. Dissecting upstream drivers—including tumor-intrinsic programs as well as paracrine cues such as cytokines, chemokines, metabolites, and tumor-derived extracellular vesicles—will be essential. A clearer mechanistic framework could inform next generation immunotherapies and improve risk stratification by anchoring prognostic interpretation to biologically grounded pathways.

Second, CD169^+^ macrophages have been reported to exert both anti- and pro-tumorigenic functions within the TME in a context-dependent manner. How these cells preserve antitumor activity within immunosuppressive niches, and why they become tumor-promoting in specific cancer settings, remain unresolved. Addressing this apparent dichotomy will require high-dimensional spatial and single-cell approaches—such as spatial transcriptomics, proteomic imaging, and cell–cell interaction inference—together with mechanistic interrogation of intracellular signaling and epigenetic states. Importantly, the identification of markers (or marker combinations) that discriminate functionally divergent CD169^+^ states will be critical for improving diagnostic specificity and prognostic precision.

Third, to reduce patient burden and enable longitudinal monitoring, non-invasive alternatives to LN or tumor biopsies are needed. Liquid biopsy is an attractive avenue: CD169 expression on circulating CD14^+^ monocytes has been proposed as a sensitive indicator of inflammatory activation in several conditions, including viral infections (e.g., COVID-19), systemic lupus erythematosus, rheumatoid arthritis, and Kikuchi disease (128–132). In parallel, recent immunoassay advances allow quantification of soluble CD169 and CD169^+^ extracellular vesicles in plasma, with reported associations to inflammatory status (133, 134). Given the immunoregulatory role of CD169 in cancer, blood-based CD169 profiling warrants systematic evaluation as a complementary platform for prognosis prediction and treatment monitoring.

In conclusion, CD169^+^ macrophages in the TME and LNs constitute a pivotal component of the host immune ecosystem and a promising prognostic biomarker axis. Future studies integrating multi-omics and spatial profiling—paired with rigorous functional validation—will be necessary to resolve their heterogeneity and context dependence. Therapeutically exploiting the TME–LN axis through CD169^+^ macrophage–informed strategies may ultimately enable more effective and personalized immunotherapy for patients with cancer.
